# Fellow Eye Macular Edema Improvement after Intravitreal Bevacizumab for Radiation Retinopathy

**DOI:** 10.1155/2015/516921

**Published:** 2015-10-08

**Authors:** Isis A. S. Brito, Leandro C. Zacharias, Sérgio Luis G. Pimentel

**Affiliations:** University of São Paulo, Rua Marieta Alves 116, 041815-260 Salvador, BA, Brazil

## Abstract

Radiation retinopathy (RR) is a progressive, chronic condition directly related to the amount of radiation administered to the retina. We report a 37-year-old patient with medulloblastoma that was treated with external beam radiation and presented to us with bilateral cystoid macular edema. He was treated with monthly bevacizumab injections only in his worst seeing eye. There was a significant improvement in his fellow eye, with marked retinal thickness reduction. Therefore, we present clinical evidence of systemic absorption and fellow eye activity of the drug (bevacizumab). One must be aware of distant side effects after intravitreal injections.

## 1. Introduction

Radiation retinopathy (RR) is a chronic and progressive condition, secondary to intraocular, encephalic, nasopharyngeal, or orbital tumor treatment. The most frequent radiation sources are external beam radiation or brachytherapy plaques [1–3].

Risk factors for RR include distance of the tumor from the optic disc, young age, and preexisting diabetes, but the total amount of radiation administered to the retina is considered to be the single most important factor. Previous data suggest that accumulated doses above 30 to 35 grays (Gy) carry a high risk of RR development [4–6].

Clinically, RR can be classified as proliferative or nonproliferative, and both stages can be related to macular edema, which is associated with worse visual outcomes [7]. Several treatment modalities, such as laser photocoagulation or intravitreal triamcinolone injections [8–10], have already been tested, but intravitreal antiangiogenic injections seem to be the most currently used therapeutic option [11–17].

## 2. Methods: Case Report

**Figure 1:**
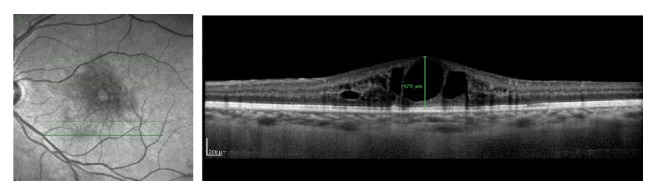
Baseline OCT of the OS shows increase in macular thickness, intraretinal cysts, and loss of foveal depression. Central macular thickness of 575 *μ*m. BCVA: 0,4 (20/50).

**Figure 2:**
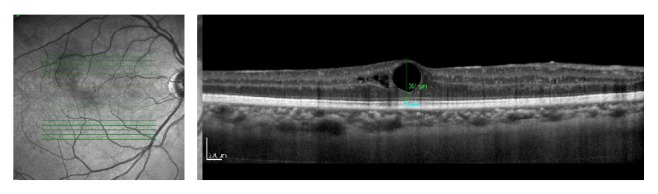
Baseline OCT of the OD shows increase in macular thickness, intraretinal cysts, and loss of foveal depression. Central macular thickness of 361 *μ*m. BCVA: 0,5 (20/40).

A 37-year-old male was submitted to 2 external beam radiation cycles for central nervous system medulloblastoma treatment in 2005 that recurred after 8 years. In 2005, the total accumulated radiation doses were 54 Gy at the posterior fossa and 36 Gy at the neuroaxis, and, in 2013, the total dose was 54 Gy. The patient came to our service complaining of bilateral progressive decrease in his visual acuity over the last 6 months. On ophthalmic examination, his best-corrected visual acuity (BCVA) was 0.5 (20/40) in the OD and 0.4 (20/50) in the OS. The biomicroscopy revealed posterior subcapsular cataract in both eyes. Fundus examination showed bilateral cystoid macular edema, without any detectable microangiopathy. Those findings were confirmed by fluorescein angiography and optic coherence tomography (OCT) (Figures 1 and 2).

**Figure 3:**
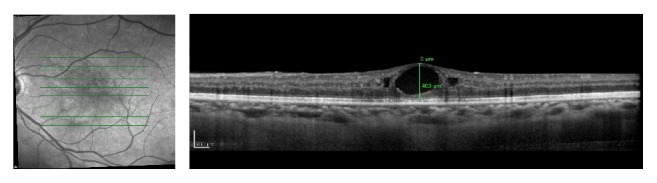
OCT of the OS 1 month after intravitreal bevacizumab injection. Partial regression of intraretinal cysts was noted. Central foveal thickness of 403 *μ*m.

**Figure 4:**
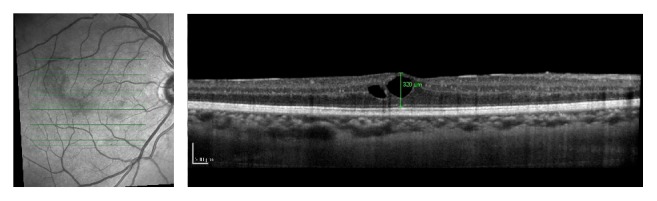
OCT of the OD 1 month after intravitreal bevacizumab injection in the fellow eye. Partial regression of intraretinal cysts was noted. Central foveal thickness of 320 *μ*m.

Due to the cystoid macular edema (CME) secondary to RR, the patient was elected to bilateral intravitreal injections of bevacizumab (1.25 mg/0.05 mL). However, the patient preferred to begin treatment only in his worst visual acuity (left eye OS). After the first intravitreal injection of bevacizumab, the cystoid macular edema showed a marked improvement in the fellow eye (OD) (Figures 3 and 4), and after the second injection it had completely resolved on that eye. The central macular thickness decreased from 361 to 217 microns, and his BCVA improved by two ETDRS lines in the OD after those two intravitreal injections in the OS (Figure 6). Therefore, the patient was treated with intravitreal bevacizumab injections only in the OS, with BCVA and macular thickness improvement after each injection and recurrence after 30 to 45 days.

## 3. Discussion

RR presents a challenge regarding effective treatment. Different from cases of diabetic retinopathy, where the initial damage occurs at the pericytes, RR is characterized by direct endothelial cell damage, leading to capillary nonperfusion and consequent poor therapeutic response [18].

Its pathogenesis involves mitosis aberrations at the endothelial cells and subsequent activation of a coagulation cascade related to the vascular endothelial damage. Clinically, those pathologic changes result in microaneurysms, telangiectasia, retinal neovascularization, vitreous hemorrhage, macular edema, and tractional retinal detachment [19–21].

CME is the first clinical manifestation of RR in most of the cases. It is found in up to 70% of patients receiving brachytherapy for choroidal melanoma after 12 months of follow-up [22]. The OCT is an important diagnostic tool, as it can diagnose macular changes on average 5 months earlier than the clinically established RR [23].

Treatment options for RR cases are still disappointing. Most of the literature agrees on anti-VEGF agents, especially when dealing with CME or retinal neovascularization [11–17].

**Figure 5:**
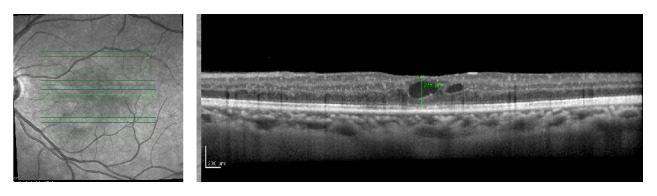
OCT of the OS after the second intravitreal bevacizumab injection. A decrease in retinal thickness was noted. Central foveal thickness of 275 *μ*m. BCVA: 0,5 (20/40).

**Figure 6:**
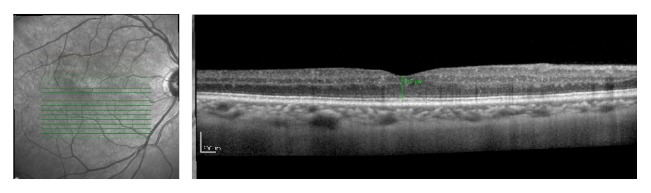
OCT of the OD after the second intravitreal bevacizumab injection in the left eye. Complete regression of intraretinal cysts was observed. Central foveal thickness of 217 *μ*m. BCVA: 0,7 (20/30).

In the current case reported, we opted for bevacizumab as the drug of choice, as previous reports support the use of this drug in cases of RR [11–15] and also due to its availability in our public service. The drug showed good efficacy regarding central macular thickness reduction and BCVA improvement at the injected eye (Figure 5). However, we also noted good anatomical and functional outcomes at the fellow noninjected eye (OD), with a 2-ETDRS-line BCVA improvement after 1 month of follow-up and total resolution of the CME after 2 intravitreal injections in the OS (Figure 6). Those data suggest systemic exposure after intravitreal bevacizumab, with fellow eye biological activity.

Long distance biological action after intravitreal bevacizumab injections has already been reported in diabetic retinopathy [24] and suggests systemic absorption and recirculation of the drug. Specifically in the case reported, that fact was advantageous, improving fellow eye function without injections, but may raise issues regarding the increase in risk for thromboembolic events [25].

## 4. Conclusion**

We report a case of CME improvement after fellow eye bevacizumab injection. To our knowledge, this phenomenon has never been previously described in cases of radiation retinopathy and reinforces the systemic exposure after intravitreous injection of this drug.
